# Study conclude that TXA in severely injured was associated with lower mortality: beware of potential confounders

**DOI:** 10.1186/s13054-021-03790-4

**Published:** 2021-11-01

**Authors:** Patrick M. Honore, Sebastien Redant, Thierry Preseau, Sofie Moorthamers, Keitiane Kaefer, Leonel Barreto Gutierrez, Rachid Attou, Andrea Gallerani, Willem Boer, David De Bels

**Affiliations:** 1 ICU Department, Centre Hospitalier Universitaire Brugmann-Brugmann University Hospital, Faculty of Medicine, ULB University, Place Van Gehuchtenplein, 4, 1020 Brussels, Belgium; 2 ED Department, Centre Hospitalier Universitaire Brugmann, Brussels, Belgium; 3 Intensive Care Department, Ziekenhuis Oost Limburgh, Campus St Jan, Genk, Belgium

With great interest, we read the recently published article by Imach et al. who concluded that tranexamic acid (TXA) use in severely injured patients was associated with a significantly lower risk of massive transfusion and lower mortality in the early in-hospital treatment period, based on matched pairing using the nationwide German Trauma Register DGU® [1]. When comparing the data between the two groups (with and without TXA), we observe that patients receiving TXA received significantly more hemostatic agents than the non TXA group (47.8% vs 44.2%, *p* value = 0.018) [1]. Hemostatic agents comprised fibrinogen, prothrombin concentrates (PPSB), calcium, factor VII or factor XIII which are all major players in coagulation after severe trauma, particularly fibrinogen [1]. All patients in the database, either with or without TXA in the matched pairs table, had an injury severity score (ISS) above 25 [1]. Life-threatening coagulopathy is present in almost all patients with an ISS > 25 with hypotension, hypothermia, and acidosis [2]. Severe hypofibrinogenemia (< 100 mg/dl) is also a frequent finding in trauma with an ISS score above 25 [2]. In patients with disrupted coagulation in severe trauma with an ISS above 25, PPSB and factors VII are crucial [3]. An advisory board comprising European trauma experts (2019) defining trauma-induced coagulopathy (TIC) in patients corresponding to those studied by Imach et al. (with an ISS > 25), recommended fibrinogen concentrate, and not fresh frozen plasma (FFP), as first-line therapy for TIC [3]. In case of further bleeding, prothrombin complex concentrate (PCC) should be given [3]. In another study, after meeting certain criteria (hemoglobin > 8 g/dl, serum fibrinogen ≥1.0 g/l, platelets > 50,000, arterial pH≥ 7.20, and body temperature ≥ 34 °C) recombinant factor VII was associated with a significantly lower mortality [4].

The fact that severe trauma patients with TXA received significantly more hemostatic agents (fibrinogen, PPSB, calcium, factor VII or factor XIII) as compared to non-TXA severe trauma patients should therefore be seen as an important potential confounding factor, reducing the solidity of the data published by Imach et al.

## Data Availability

Not applicable.

## Abbreviations

TXA: Tranexamic acid
PPSB: Prothrombin concentrates
ISS: Injury severity score
TIC: Trauma-induced coagulopathy
FFP: Fresh frozen plasma
PCCs: Prothrombin complex concentrates
BP: Blood pressure
MTP: Mass transfusion protocol
