# Supplementary material for: Study conclude that TXA in severely injured was associated with lower mortality: beware of potential confounders
Source: Crit Care. 2021 Nov 1;25:379. doi: 10.1186/s13054-021-03790-4 (PMC8561903; doi:10.1186/s13054-021-03790-4)
Supplement: Related texts

# Reply: Response to letter: Study conclude that TXA in severely injured was associated with lower mortality: beware of potential confounders

**Authors:** Sebastian Imach, Arasch Wafaisade, Rolf Lefering, Andreas Böhmer, Mark Schieren, Victor Suárez, Matthias Fröhlich, TraumaRegister DGU

**Affiliations:** 4 grid.412581.b 0000 0000 9024 6397 Department of Trauma and Orthopedic Surgery, Cologne-Merheim Medical Center (CMMC), University Witten/Herdecke, Cologne, Germany; 5 grid.412581.b 0000 0000 9024 6397 Institute for Research in Operative Medicine (IFOM), University Witten/Herdecke, Cologne, Germany; 6 grid.412581.b 0000 0000 9024 6397 Department of Anaesthesiology and Intensive Care Medicine, Cologne-Merheim Medical Center (CMMC), University Witten/Herdecke, Cologne, Germany; 7 grid.6190.e 0000 0000 8580 3777 Department II of Internal Medicine (Nephrology, Rheumatology, Diabetes and General Internal Medicine) and Center for Molecular Medicine Cologne, University of Cologne, Faculty of Medicine, Cologne, Germany; 8 grid.411097.a 0000 0000 8852 305X University Hospital Cologne, Cologne, Germany; 9 Committee on Emergency Medicine, Intensive Care and Trauma Management (Sektion NIS) of the German Trauma Society (DGU), Berlin, Germany

We would like to thank Honore et al. for their critical appreciation of our work. They have legitimately dealt with the potential main confounder of our study [1]. The aim of our work was to investigate the effects of preclinical administration of TXA in severely injured patients (ISS TXA 32.4 SD 14.7, Control 32.0 SD 14.7) in a developed healthcare system. In those systems, the sole administration of TXA is not expected to be the only therapeutic measure that is taken in the care for a severely injured patient. The patients in the study did not all meet the conditions for life-threatening coagulopathy defined by Haas and Cushing [2]. Only 24.6% in the TXA group have been in shock ((BP ≤ 90 mmHg) at hospital admission. Mean base excess as a parameter representing potential acidosis was − 4.5 mmol/l (SD 6.1) in TXA (Control − 4.3(6.3)). We had no data concerning body temperature. Consequently, the use of coagulation factors does not seem to be mandatory for every patient because of an expected life-threatening coagulopathy. However, we agree that a trauma-induced coagulopathy (TIC) should be treated by hemostatic agents [3]. This database represents the standard of care in Germany with Fresh Frozen Plasma being a part of it. In the literature, at least indications can be found in the case of mass transfusions [5].

The previously cited work by Černý et al. calls for the avoidance of mass transfusion protocols (MTP) as the main goal of therapy and recommends the immediate administration of TXA. In your study, preclinical administration of TXA seems to trigger a more comprehensive resuscitation approach in the early hospital phase since TXA group received more i.v. fluids, blood transfusion were started more frequently and more hemostatic drugs were administrated. The trigger for this approach remains unclear, as the collective was matched for the usual triggers (BP, trauma mechanism, injury severity). This approach undoubtedly begins with the preclinical administration of TXA. The suspected risk of bleeding can be an explanation for this. The search for further measurable triggers of therapy should be a future research interest but cannot be answered by our database.
